# Recent Advances in the Surface Functionalization of PLGA-Based Nanomedicines

**DOI:** 10.3390/nano12030354

**Published:** 2022-01-22

**Authors:** Mazen M. El-Hammadi, José L. Arias

**Affiliations:** 1 Department of Pharmacy and Pharmaceutical Technology, Faculty of Pharmacy, University of Seville, 41012 Seville, Spain; mazenhammadi@us.es; 2 Department of Pharmacy and Pharmaceutical Technology, Faculty of Pharmacy, University of Granada, 18071 Granada, Spain; 3 Institute of Biopathology and Regenerative Medicine (IBIMER), Center of Biomedical Research (CIBM), University of Granada, 18100 Granada, Spain; 4 Biosanitary Research Institute of Granada (ibs.GRANADA), Andalusian Health Service (SAS), University of Granada, 18071 Granada, Spain

**Keywords:** active drug targeting, ligand-mediated targeting, nanoparticle, passive drug targeting, PLGA, stealth coating, surface functionalization

## Abstract

Therapeutics are habitually characterized by short plasma half-lives and little affinity for targeted cells. To overcome these challenges, nanoparticulate systems have entered into the disease arena. Poly(d,l-lactide-*co*-glycolide) (PLGA) is one of the most relevant biocompatible materials to construct drug nanocarriers. Understanding the physical chemistry of this copolymer and current knowledge of its biological fate will help in engineering efficient PLGA-based nanomedicines. Surface modification of the nanoparticle structure has been proposed as a required functionalization to optimize the performance in biological systems and to localize the PLGA colloid into the site of action. In this review, a background is provided on the properties and biodegradation of the copolymer. Methods to formulate PLGA nanoparticles, as well as their in vitro performance and in vivo fate, are briefly discussed. In addition, a special focus is placed on the analysis of current research in the use of surface modification strategies to engineer PLGA nanoparticles, i.e., PEGylation and the use of PEG alternatives, surfactants and lipids to improve in vitro and in vivo stability and to create hydrophilic shells or stealth protection for the nanoparticle. Finally, an update on the use of ligands to decorate the surface of PLGA nanomedicines is included in the review.

## 1. Introduction

**Figure 1:**
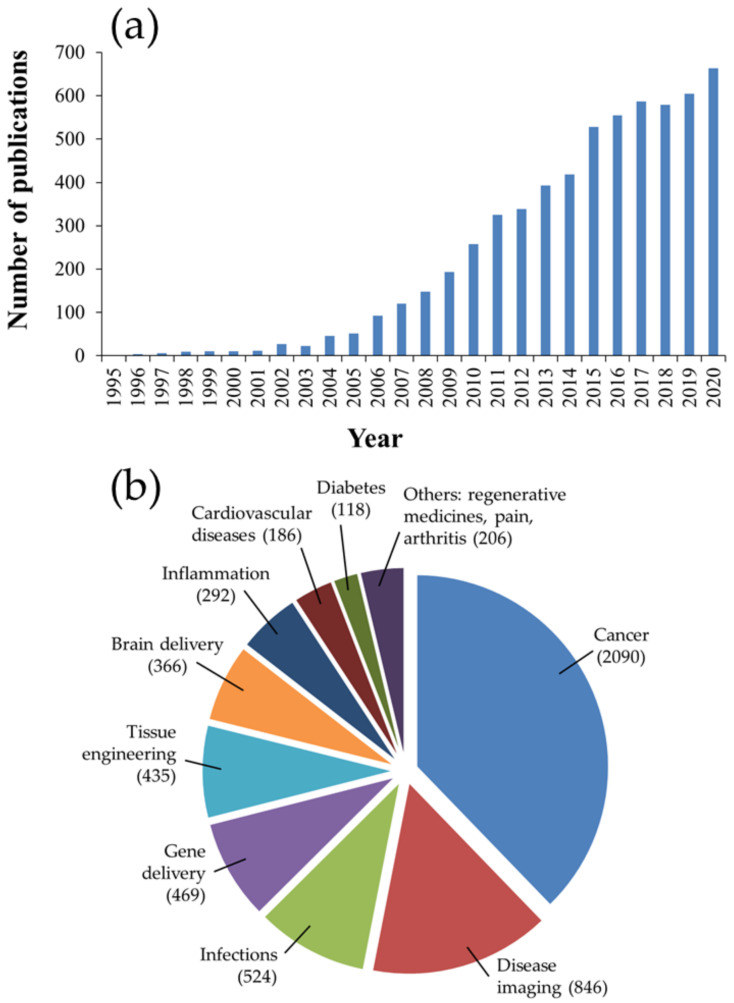
PLGA-related publications on PubMed (accessed on 15 October 2021) between 1995 and 2020. (**a**) Total publications (search keywords used were “PLGA” and “nanoparticles”). (**b**) Publications on PLGA NPs according to biomedical applications.

In recent decades, nanomedicine has received significant interest for its biomedical applications, including prevention, diagnosis, and treatment of diseases. Nanomedicine involves the engineering of nano-sized or nanostructured systems with unique physiochemical properties and physiological qualities [1,2]. Among therapeutic nanosystems, polymer-based nanoparticles (NPs) offer many advantages, such as improved drug efficacy and delivery, enhanced drug solubility, modified pharmacokinetic profile, a longer blood circulation time, and minimal toxicity, to name a few [3]. Polymers, which can be synthetic, such as poly(d,l-lactide) (PLA), poly(d,l-lactide-*co*-glycolide) (PLGA) copolymers, poly (*ε*-caprolactone) (PCL) and poly(amino acids), or naturally occurring, such as alginate, chitosan (CS) and gelatin, have been widely used as NP-forming biomaterials due to their diverse characteristics, flexibility of design, synthesis, functionalization and, more importantly, favorable safety profile and low toxicity. Amongst these polymers, PLGA copolymers, which are approved for medical application by the United States Food and Drug Administration (FDA) and the European Medicines Agency (EMA), is a synthetic versatile copolymer that enjoys desirable biocompatibility and biodegradability, as well as unique physical and chemical properties, making it one of the most favored and efficient polymers used in drug delivery [4,5,6,7,8,9,10]. A brief examination of the number of publications on PubMed using the keywords “PLGA” and “nanoparticles” reveals its overwhelming growth, by hundreds of folds, in the last 25 years, as shown in Figure 1.

In this review, recent advances in the surface engineering strategies utilized for PLGA-based NPs are addressed. The background of the properties and biodegradation of the copolymer is also provided, as well as a brief discussion on the methods of fabrication and in vivo fate of PLGA NPs.

## 2. Physicochemical Properties of PLGA

PLGA is a synthetic copolymer composed of two monomers: lactic acid (LA) and glycolic acid (GA). It is mainly synthesized through the random ring-opening copolymerization of the cyclic dimers (1,4-dioxane-2,5-diones) of GA and LA. An ester linkage is formed during copolymerization connecting the two acids, consequently producing an aliphatic linear polyester [4,9,10]. The monomer ratio is usually used to identify the polymer’s form. For example, PLGA 50:50, which is broadly employed in drug delivery [11,12,13,14], means a PLGA copolymer with 50% of LA and GA, respectively. The most common LA:GA ratios employed in research are 50:50, 65:35, 75:25 and 85:15 (LA:GA ratio) [15].

The physical properties of different commercially available forms, such as the degree of crystallinity, molecular weight (M_W_), and melting point, significantly affect the polymer’s mechanical strength, its usability as a drug nanocarrier, and its stability (Table 1).

One notable feature is the PLGA crystallinity, which directly influences its mechanical strength, swelling behavior, hydrolysis ability and, subsequently, its biodegradation rate [16]. The polymer’s crystallinity is correlated with the type and molar ratio of GA and LA in the copolymer chain [16]. Copolymers synthesized using poly(d,l-lactide) and poly(glycolide) are amorphous, whereas those originated from poly(l-lactide) and poly(glycolide) show crystalline characteristics. Furthermore, PLGAs with <70% of poly(glycolide) are also amorphous.

Similarly, the solubility of PLGA also varies according to the M_W_ and monomer proportions. LA is less hydrophilic than GA; therefore, LA-rich copolymers are more hydrophobic. However, the hydrophobicity of PLGA is reduced with decreasing M_W_ and, at 1100 Da, it becomes water-soluble [17].

In addition, the glass transition temperature (T_g_) of PLGA is also dependent on the monomer ratio and is reduced with the decrease in M_W_ and LA content [16]. T_g_ refers to the temperature at which the polymer converts from the rubbery state (temperatures above T_g_) to the glassy state (temperatures under T_g_). Since PLGA copolymers feature a T_g_ in the range of 45 to 55 °C, which is above the physiological temperature (37 °C), they are normally characterized by the mechanical strength and stiffness of the glassy polymer, with sufficient strength for formulation development.

In brief, selecting the suitable PLGA for the desired application is subject to several factors, among which monomer ratios and M_W_ are of utmost importance as they determine the major characteristics of the copolymer.

## 3. Biodegradation of the PLGA Copolymer

PLGA experiences hydrolytic degradation, which is triggered immediately upon contact with aqueous solutions and generates acids, which in return catalyze the hydrolysis [17]. Bulk erosion has been found to be the main degradation pathway for PLGA, although polyester can also suffer surface erosion. Generally, the biodegradation of PLGA occurs in several steps. It is initiated by the hydration of the amorphous region and the disruption of hydrogen bonds and van der Waals forces, followed by the hydrolysis of ester bonds in the polymer backbone, which results in a remarkable reduction in the M_W_. This process continues with the cleavage of covalent bonds with a further reduction in M_W_, accompanied by a rapid loss of mass and the formation of soluble oligomeric products. Finally, the segregated fragments are cleaved into soluble molecules [7,9].

Polymer composition, degree of crystallinity, T_g_, average M_W_, size and shape of the NP matrix, as well as pH are also factors that can affect the degradation of PLGA in vivo. Overall, higher hydrophilicity of the polymer backbone or end groups (greater ratios of GA), lower crystallinity, smaller average M_W_, lower size of the final NP (i.e., greater surface area to volume ratio), and both strongly alkaline and acidic media lead to an increase in the degradation rate of PLGA polymers [9,18]. For example, a PLGA with a 40:60 ratio (LA:GA) and M_W_ of 5 kDa degrades faster than a polymer with a 50:50 ratio and M_W_ of 15 kDa. In this context, the impact of the M_W_ of four 50:50 (LA:GA) PLGA copolymers with different M_W_ of 14.5, 45, 85, and 213 kDa on the degradation of estradiol-loaded NPs was explored [19]. It was found that increasing the M_W_ of the PLGA chain led to a significant decrease in the rate of NP degradation and drug release. Thus, the percentage of released drug under physiological conditions on day 18 decreased from the 95% achieved by NPs prepared using low M_W_ copolymer down to 23%, as reported by those formulated using high M_W_ PLGA. Similarly, a recent report showed that even with a few kDas (10.2 and 10.3 vs. 4.7 kDa) a considerable difference in the rate of drug release and polymer degradation could be observed [20]. In addition, it has been observed that the higher the M_W_ of PLGA (6, 14.5, and 63.6 kDa), the larger the size of NPs loaded with paclitaxel (122 ± 3, 133 ± 2, and 160 ± 2 nm, respectively) and of NPs without paclitaxel (117 ± 2, 132 ± 2, and 159 ± 3 nm, respectively) [21]. In addition, some findings suggest that the amounts and properties of loaded drugs can also determine the rate of matrix degradation and body fate of PLGA-based particles [7]. Based on these facts, a proper selection of the polymer’s properties, such as M_W_ and/or molar ratios of the monomers, can be used to modulate the PLGA degradation time and, hence, control the drug release rate, which can be prolonged for up to several months.

Following the hydrolysis of the polymer inside the body, the removal of the final PLGA products from the body takes place in two major ways, namely, renal excretion and respiration. Thus, some of the original monomers (LA and GA), which are also byproducts of various metabolic pathways, are excreted unchanged in the urine. The rest are easily metabolized as endogenous and non-toxic derivatives through the Krebs’ cycle and, consequently, eliminated as carbon dioxide and water [22,23]. While the hydrolysis pathway is broadly believed to be solely responsible for PLGA biodegradation, the potential contribution of enzymatic degradation remains unclear.

Accordingly, the biodegradation of PLGA and the complete removal of its products make its use highly safe with ignorable systemic toxicity.

## 4. Preparation of PLGA NPs

PLGA NPs can be produced using several top-down techniques, in which the NPs are fabricated from the pre-formed copolymer. Some of the most widely investigated techniques for the preparation of PLGA NPs, emulsion evaporation, nanoprecipitation or solvent displacement, solvent diffusion, and salting out are discussed in this Section (Table 2) [5,6,24,25,26].

### 4.1. Emulsion Evaporation Method

The emulsification method is considered the first technique employed to produce polymeric NPs from preformed polymers. In this method, a volatile, non-water miscible solvent (e.g., ethyl acetate, chloroform, methylene chloride), in which the polymer is dissolved, is emulsified with an aqueous solution containing a surfactant to stabilize the emulsion. Emulsification is performed using high shear force to minimize the diameter of the emulsion droplet, which is directly related to the final NP size. Next, the volatile organic solvent is removed by evaporation, which results in NP formation [41]. The influence of different experimental parameters on the particle size, zeta potential (*ζ*), drug loading efficiency and drug release of PLGA NPs prepared using this method has been reported in previous research [27,28,29,30]. In general, the major influential formulation factors are the initial concentrations of polymer, surfactant and drug. This technique is relatively non-toxic, generates controllable and very small particles, is easy to scale up, and can be used to encapsulate both water-soluble and water-insoluble drugs [42]. Hydrophobic compounds can be loaded using o/w emulsion, whereas hydrophilic compounds can be entrapped by the formation of a w/o/w double emulsion. However, standardization may be required for each specific drug, and the stability of some drugs may be affected by the high energy employed during emulsification [42].

### 4.2. Nanoprecipitation Method

Nanoprecipitation, also known as the solvent displacement method, involves the addition of a polar, water-miscible solvent, mostly acetone, ethanol, hexane, methylene chloride or dioxane, in which PLGA is dispersed into an aqueous phase using low-energy mixing [43]. Based on the so-called Ouzo effect, spontaneous emulsification takes place and, driven by the rapid diffusion of the organic solvent into water, the hydrophobic polymer solutes become dispersed in the aqueous phase (antisolvent) [44]. Consequently, the polymer precipitates as nano-sized particles following a four-step mechanism, which includes supersaturation, nucleation, growth by condensation, and growth by coagulation [43]. The solvent is then removed by evaporation, extraction, or a combination of both. The size of PLGA NPs fabricated by nanoprecipitation is dependent on the polymer concentration [31]. By altering the polymer amount, PLGA NPs with two different particle sizes, 230 and 160 nm, were produced by nanoprecipitation. While larger NPs were found to feature higher encapsulation efficiency, smaller NPs appeared to be more effective in vitro when both NPs were applied at the same weight concentration [32]. Furthermore, it has been shown that parameters such as a high concentration of polymer in the organic solution, organic solvent of very low diffusion coefficient in water, and high salt concentration encourage the formation of large PLGA NPs and, therefore, should be avoided [33]. Nanoprecipitation is advantageous because it is simple, rapid, avoids the use of toxic solvents, generates NPs with narrow size distribution, and does not need the use of high energy [45]. However, the method is not adequate for hydrophilic drugs due to diffusion to the aqueous phase, as well as the fact that solvent removal can be time-consuming, particle size is significantly affected by the polymer concentration, and controlling particle growth can be problematic [42].

### 4.3. Solvent Diffusion Method

The distinction between solvent diffusion and emulsion evaporation methods is based on the selection of solvent. In the former method, the organic solvent, which must be partially miscible in water (e.g., benzyl alcohol, propylene carbonate, ethyl acetate), is emulsified with an aqueous solution of a suitable surfactant [e.g., sodium dodecyl sulfate, polyvinyl alcohol (PVA)] under stirring. NP formation is induced by the solvent diffusion and the counter diffusion of water into the emulsion droplets [42]. It was demonstrated that the emulsification solvent diffusion method could be used for the fabrication of PLGA NPs with highly reproducible sizes (between 50 and 400 nm) and a relatively narrow polydispersity [34]. For the preparation of PLGA NPs, it is also common to use a binary solvent mixture consisting of water-immiscible and water-miscible solvents (e.g., methylene chloride-acetone) [35,36]. In this method, highly toxic solvents and high stress shear are avoided. However, the process requires large quantities of water and long agitation time, and particle size is significantly influenced by polymer concentration (when low-speed agitation is used). While the method is suitable for loading hydrophobic molecules, hydrophilic drugs tend to migrate to the aqueous phase and thus are associated with low drug entrapment efficiencies [24].

### 4.4. Salting Out Method

The salting-out process is induced by using a high concentration of salts that are insoluble in the organic solvent, e.g., magnesium chloride hexahydrate, magnesium acetate tetrahydrate, or sucrose [5,6]. The organic phase, which should be water-miscible (e.g., acetone or tetrahydrofuran), is emulsified with the aqueous phase under high shear stress agitation. Unlike the emulsion diffusion method, the organic solvent cannot diffuse because of the presence of salts. However, the ionic strength is reduced by the dilution of the o/w emulsion with water, under mild stirring, which leads to the migration of the solvent to the outer aqueous phase and the formation of the PLGA NPs [37,38,39]. Finally, the salting out agent is removed by cross-flow filtration or centrifugation. The process is low time-consuming and does not require high stress shear. Using a method combining the salting out and emulsification techniques, the encapsulation of meloxicam in PLGA NPs with varying Mw (5 to 15 and 40 to 75 kDa) was investigated. The study concluded that the polymer with higher Mw produced the most physically stable NPs [41]. In this method, heat-sensitive drugs (since no heating is required) and high concentrations of PLGA can be employed [46]. However, it is not favorable for lipophilic drugs and requires a purification step to eliminate the salting out agent which may be time-consuming. Besides, the solvents used, although not highly toxic, may be explosive [24].

### 4.5. Emerging Production Methods

With the aim of enhancing the productivity, reproducibility, and scalability of PLGA NPs, several production methods, such microfluidic technology [12], supercritical fluid technology [47], and membrane-assisted strategies [48], have recently been investigated. Among these approaches, particle replication in non-wetting templates (PRINT), a soft lithography platform, enables the ability to control size and shape, independent of process variables, and the generation of truly monodisperse particles [49]. The method is not limited to sphere NPs but has been used to produce other shapes of PLGA NP, such as needle-shaped [50] and cylindrical-shaped [51] particles; it is scalable, and allows the easy encapsulation of a wide range of cargos, including hydrophilic or hydrophobic therapeutics. The method involves the preparation of a thin film of PLGA and the drug being used, spread on a sheet of perfluorinated polyether elastomer. The film is then heated while placed in contact with a mold, resulting in the flow of the polymer into the cavities of the mold. Next, the polymer is solidified and the NPs are collected from the mole to any flat surface or to an excipient layer via reheating in contact with the desired surface [52].

Furthermore, microfluidic technology offers an interesting alternative in the preparation of PLGA NPs. This approach uses microfluidic chips and is based on the laminar flow of the liquids confined in the microchannels. The restricted volume, stream flow rates, and chip design enable the regulation of the mixing of the polymer solution with the antisolvent phase [12]. Close control of these parameters has been shown to improve the tunability of the NP properties, allowing the optimization of the particle size and polydispersity index [53,54]. Although this technology is promising, its industrial implementation still requires the development of robust protocols for the fabrication of well defined particles.

## 5. Biological Fate of PLGA NPs

The rationale behind using NPs in drug delivery is to carry and protect the therapeutic payloads and ultimately deliver them to the sites of action. To achieve this objective, it is crucial that the administered NPs overcome the multiple biological barriers faced during their journey within the human body.

One major challenge for systemically administered PLGA-based NPs is the clearance by the mononuclear phagocyte system (MPS). This system, comprised of dendritic cells, blood monocytes, granulocytes, and tissue-resident macrophages in the liver, spleen, and lymph nodes, imposes an effective defense mechanism through the elimination of exogenous materials and foreign pathogens [55]. Due to the highly fenestrated endothelia specifically encountered in organs associated with the MPS, circulating materials are screened rigorously. This is of particular importance for PLGA NPs, which can diffuse across the permeable vascular endothelia in these organs, resulting in rapid clearance from the bloodstream. Additionally, PLGA NPs circulating in blood are subjected to opsonization, a process that involves the surface adsorption of serum proteins, known as opsonins, which include complement compounds, immunoglobulins, fibronectin, and apolipoproteins [56]. The adsorption of opsonins to the particle surface can occur through a number of events, including hydrophobic interactions, and, to a lesser extent, electrostatic interactions and hydrogen bonding [57,58]. As a result, opsonized PLGA NPs are more readily recognized and captured by cells in the MPS, which overexpress a variety of opsonin-recognizing receptors, including complement, Fc, and fibronectin receptors [56]. Furthermore, it has been shown that serum proteins can rapidly interact with circulating PLGA NPs, forming a protein corona composed of up to hundreds of different proteins. Thus, the formation of the protein corona was found to promote the non-specific uptake of NPs by cells encountered during their circulation, including endothelial cells [58]. The complete removal of conventional PLGA NPs from the bloodstream has been estimated to occur within 10 min of systemic administration, regardless of the composition of the particle [58,59]. As a consequence, not only the circulation time of PLGA NPs is minimized, but the targeting capabilities of NPs functionalized for targeting specific cells is also clearly undermined.

As for the cellular internalization of PLGA NPs, similar to other polymeric NPs, two main mechanisms have been identified, including clathrin-mediated endocytosis and fluid-phase pinocytosis. Consequently, the NPs are released into the cytosol through endosomal escape resulting from the temporary destabilization of the endosomal membrane [7].

**Figure 2:**
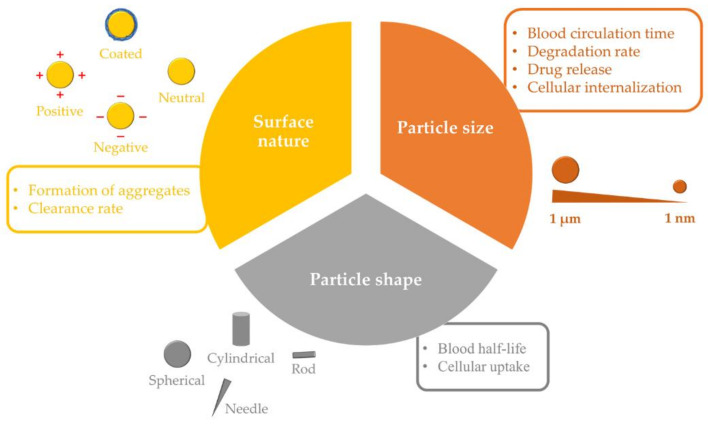
Effects of the physical properties of PLGA NPs on their in vivo behavior.

The characteristics of PLGA NPs, such as particle size (100 to 500 nm) and surface nature (charge and hydrophilicity), exert a considerable influence on their fate in biological systems (Figure 2). These features largely determine the possible physicochemical interactions between NPs and biological compounds, making them of high importance for the design and formulation of the nanosystem [60].

While larger-sized PLGA NPs (>300 nm) are generally associated with increased drug loading capacity, PLGA NPs with a relatively small size range (50 to 100 nm) demonstrate longer circulation times and possess a greater ability to overcome biological barriers and reach the site of action. Furthermore, it has been demonstrated that the effects of particle size on cellular internalization are greatly influenced by NP composition and its behavior in biological media [61,62]. In addition, size has been linked to degradation rate and drug release kinetics, with a tendency of smaller PLGA particles to degrade at a faster rate, resulting in a relatively rapid drug release, in comparison with lager PLGA particles [18,63]. Moreover, PLGA with higher M_W_ has been found to increase the mean diameter of resulting NPs. This is because the polymer M_W_ can affect the viscosity of the organic phase, thereby decreasing the net shear stress and increasing the particle size [64]. It is worth mentioning that small NPs (<10 nm) can also be cleared by renal excretion. However, this is unlikely to influence the clearance of PLGA NPs designed for drug delivery, which typically have higher sizes [65].

Another important factor that affects NPs’ in vivo fate is their composition and surface nature. While neutral hydrophobic NPs readily aggregate in aqueous solutions owing to van der Waals and/or hydrophobic forces and are, therefore, rapidly cleared by the MPS, charged NPs, both positive and negative, are governed by repulsive forces that render them relatively stable in aqueous conditions as long as the ionic strength is low [65]. However, in solutions with high ionic strength, such as blood, NPs may be neutralized by counterions, increasing the possibility of aggregation. Large aggregates of systemically administered NPs have been shown to be physically entrapped in the first capillary bed in the lung, resulting in rapid elimination from the circulation, as reported with positively charged polyplexes [polymer-deoxyribonucleic acid (DNA) complexes] [66]. Furthermore, the surface charge can interfere with the ability of NPs to cross biological membranes; thus, the negative charge of PLGA NPs may limit their cellular internalization. For example, when cellular uptake of negative (*ζ* ≈ −25 mV) and positive (modified with hexadecyltrimethylammonium bromide, CTAB; *ζ* ≈ +15 mV) PLGA NPs that feature similar particle sizes (≈80 nm) was investigated in vitro, the positively charged NPs were easily internalized in both L5178Y and TK6 cell lines (95.5% and 41.1% of positive cells, respectively), while very low penetration was detected with negatively charged PLGA NPs (1.8% and 7.8% of positive cells, respectively) [67].

Particle shape is another influential element in the biological performance of PLGA NPs. In comparison with their spherical counterparts, and due to their geometries, non-spherical NPs have been found to produce superior biological performance, including extended blood circulation times, reduced immune clearance, and improved tumor accumulation in solid malignancies [68,69]. They also show a unique ability to bind to cells and to be internalized rapidly [70]. Thus, PLGA NPs of various shapes have been formulated and explored for their biodistribution, cellular uptake and in vivo drug delivery performance. Two cylindrically shaped docetaxel-loaded PLGA NPs were fabricated using PRINT technology. They were 80 × 320 nm (diameter × height), and 200 × 200 nm (diameter × height). The NPs improved plasma pharmacokinetics and tumor delivery compared to the clinical formulation of docetaxel: the volume of distribution, plasma exposure and tumor exposure were ≈18–33 fold lower, ≈20 fold higher, and ≈50 to 75% higher compared to docetaxel [51]. In addition, the particle exhibited lower clearance by organs of the MPS in comparison with the larger NPs. Similarly, rod-shaped, docetaxel-loaded PLGA NPs with a diameter of 215 nm and a low polydispersity index value of 0.05 were fabricated using PRINT technology. The pharmacokinetic analysis demonstrated the ability of these NPs to increase the drug circulation time and provided similar docetaxel exposure to tumors compared to the free chemotherapeutic [50]. Furthermore, needle-shaped PLGA particles were prepared using the film-stretching method. In this method, spherical PLGA particles embedded in a PVA film were stretched using a film-stretching apparatus in one dimension. Next, the film was dissolved in water and the nanoneedles recovered. The needle-shaped PLGA NPs were found to be more efficient at crossing endothelial cell membranes in vitro and delivering drugs such as small interfering ribonucleic acid (*si*RNA) into the cellular cytoplasm in comparison with their spherically shaped counterparts. This resulted in a 150% increase in internalization with threefold higher silencing effectiveness compared to the spherical analogs. Although the mechanism of needle-shaped PLGA particle internalization is not yet fully understood, three possible pathways were proposed, including endocytosis, direct delivery by puncturing the cell, and/or membrane portion [71]. However, following endocytosis, needle-shaped PLGA NPs can damage the lysosomal membrane, inducing lysosome disruption. This damage activates the signaling pathways for cell apoptosis, leading to DNA fragmentation and cell death [72].

## 6. Surface Modification Strategies

The main intrinsic disadvantages associated with PLGA-based NPs, particularly their rapid clearance from blood circulation, limit their biological half-life, their ability to reach the site of action and, hence, their effectiveness [73]. In addition, unmodified PLGA NPs do not possess cell recognition capabilities, making them unselective, i.e., unable to recognize diseased cells/tissues. Instead, PLGA NPs tend to distribute to a few tissues, including liver, bone marrow, lymph nodes, and spleen, thereby limiting their ability to target other organs [74]. Furthermore, the passive accumulation of PLGA NPs in tumor tissues due to the enhanced permeability and retention (EPR) effect may not occur at sufficient levels to produce efficient therapeutic effects. Furthermore, because of their negative surface charge, PLGA NPs are highly internalized by phagocytic cells, whereas their uptake by other cells is relatively poor [75]. Increasing evidence, based on studies of the role of surface modification, suggests that the incorporation of surface-modifying agents can substantially influence the performance of NPs and significantly increase their blood circulation time [9,76,77].

**Figure 3:**
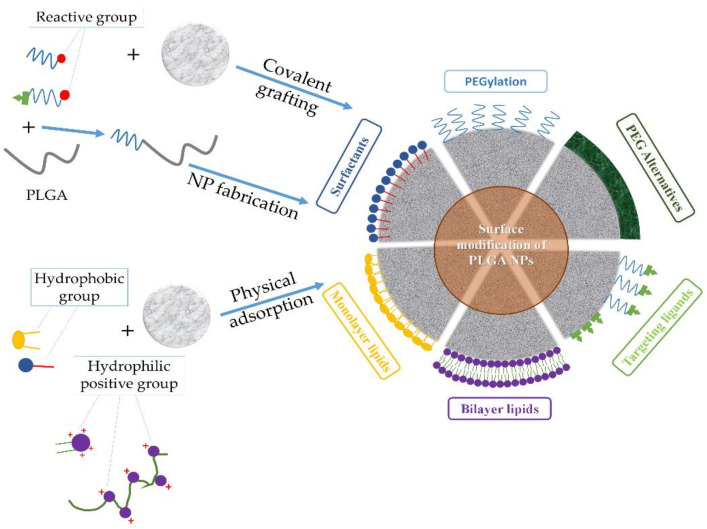
Different strategies used in the surface modification of PLGA NPs.

Addressing the drawbacks of PLGA-based nanosystems necessitates the precise engineering of the NP to alter the unfavorable properties and produce new features. To this end, surface functionalization has been adopted as a major strategy to generate PLGA NPs with desirable characteristics and enhanced in vivo efficiency. Thus, issues such as opsonization, blood circulation time, adverse drug effects, and targeted therapy of PLGA-based nanosystems can all be dealt with using surface functionalization techniques, such as PEGylation [coupling of poly(ethylene glycol) (PEG) chains], surfactant- or lipid-coating, and surface decoration with cell-targeting ligands (Figure 3 and Table 3). However, to the best of our knowledge, during the last decade, few clinical trials have evaluated the real possibilities of these PLGA-based nanosystems (Table 4).

### 6.1. Surfactants

Due to the hydrophobic nature of PLGA NPs, surfactants are used in their fabrication process to improve their colloidal stability. Surfactants function by decreasing the surface tension of particles at interfaces with the non-solvent, thus preventing particle aggregation. Among the commonly employed surfactants in the preparation of PLGA NPs, PVA is known to produce NPs with reduced size and uniform distribution [42]. However, there are concerns about the safety of this polymer as subcutaneous and intravenous administration are associated with hypertension, anemia, and central nervous system depression in animals [78]. Other surfactants used in the nanofabrication of PLGA particles include poloxamers (non-ionic polypropylene oxide and polyethylene oxide tri-block copolymers, also known as, e.g., Pluronic^®^ F-68, Pluronic^®^ F-127), polysorbates (e.g., Tween^®^ 20, Tween^®^ 80), sodium cholate, and vitamin E TPGS (d-*α*-tocopheryl polyethylene glycol 1000 succinate) [79]. The type and concentration of the surfactant can determine not only the particle’s physical stability but also other important parameters, such as the drug’s loading and release capacities, pharmacokinetics, and cellular uptake [27,80,81]. For instance, in a study that compared surface coating of PLGA NPs with either PVA or Pluronic^®^ F-127, different internalization efficiencies were found when the NPs were examined in vitro, with greater uptake of Pluronic^®^-coated NPs by breast cancer cells in comparison with PVA-modified PLGA NPs [78]. In addition, it has been suggested that the use of polysorbates can improve PLGA particle circulation time and the permeation of the blood–brain barrier (BBB) [8]. Combining more than one surfactant may also be considered. For example, PLGA NPs formulated using a blend of Span^®^ 60 and Pluronic^®^ F-68, loaded with paclitaxel and administrated systemically in mice, demonstrated an increased circulation time, increased accumulation in lung and brain tissues, and a greater reduction in tumor volume (44.6%) in comparison with the free drug (24.4%), with no observed change in the toxicity of the anticancer agent [13]. Although the mechanism through which this combination of surfactants can enhance PLGA NP stability and therapeutic efficiency still require more in-depth exploration, it is anticipated that it would combine the effects of used surfactants and enable the use of lower concentrations, thus reducing potential toxicity.

### 6.2. PEGylation of PLGA

PEG, a non-ionic synthetic polymer, is widely used in the biomedical field owing to its hydrophilicity and safety in humans; it is classified as “Generally Regarded as Safe” (GRAS) by the FDA. PEG is commercially available in a wide range of M_W_, from 300 Da up to 100 kDa; despite being non-biodegradable, it is highly biocompatible and, at a lower M_W_, it is excreted unchanged in the kidney without accumulation in the body’s tissues [82].

Owing to their hydrophilicity, aqueous solubility, and non-ionic nature, PEG coatings on PLGA NPs increase the surface hydrophilicity and provide it with a near-zero *ζ*, thus shielding the surface from aggregation and opsonization, prolonging blood circulation time, and improving pharmacokinetics [48,83,84]. It has been shown that a higher density of PEG surface coating on the nanosystem leads to increased biological half-life [85]. Furthermore, the length of the PEG chain can influence the size of PLGA NPs. In an example, it was found that shortening the PEG chain from 20 to 2 kDa reduced the particle size from ≈570 down to ≈180 nm [86].

**Figure 4:**
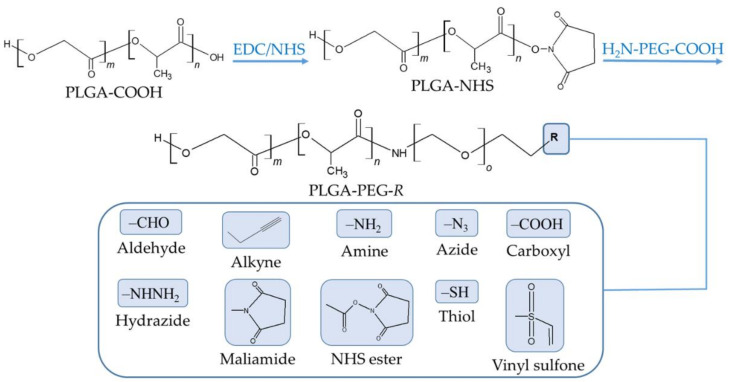
Schematic representation of the synthesis of a PLGA-PEG conjugate using a carbodiimide coupling reaction. The conjugate can be further functionalized using PEG derivatives with various terminal groups.

The PEGylation of PLGA NP surfaces is achieved by using one of three typical coating approaches, including the physical adsorption of PEG chains to NPs through electrostatic or hydrophobic interactions, the rafting of PEG onto the surface of NPs by forming a stable chemical bond, and the conjugation of PEG to the PLGA chain to produce a PLGA-PEG co-polymer that is then used to fabricate NPs [87]. PEGylated PLGA NPs can be further functionalized with targeting ligands using PEG derivatives with various terminal groups (Figure 4).

In addition, PEG modification has been shown to enhance the diffusion of PLGA NPs through mucus, which is of particular importance for effective local delivery of therapeutics [88,89]. For instance, it was shown that the PEGylation of PLGA NPs improved uptake of larger NPs (≈335 to 400 nm) over smaller non-PEGylated NPs (≈300 nm) in an in vitro intestinal barrier model [90]. Furthermore, PEG-PLGA conjugates can be exploited to produce self-assembling micelles and vesicles. Achieving these desirable benefits is largely dependent on the nature of the PEG–surface bond and its M_W_ and density. In general, covalent linking (rather than simple physical adsorption to the NP surface), higher M_W_, and higher surface density of PEG are key factors to increase the circulation time of PEGylated NPs [22,65]. However, PEGylation is associated with several limitations. One major challenge is the optimization of PEG’s M_W_ to attain prolonged systemic circulation without compromising drug activity. In addition, due to its non-biodegradable nature, PEG may not be excreted efficiently in the kidneys, thereby increasing its potential accumulation in the liver and cellular lysosomes of normal tissues, which in turn may lead to the occurrence of macromolecular syndrome. Furthermore, while the enlargement of PLGA NPs caused by the PEG chains extended on the surface may unexpectedly promote MPS uptake, resulting in rapid elimination from circulation, the hydrophilicity of these chains may also reduce cellular uptake [91]. Moreover, the issue of PEG immunogenicity has recently been under the spotlight. Specific anti-PEG antibodies can be produced as a response to previous treatment with PEGylated drugs or the consumption of products containing PEG, and can cause accelerated blood clearance, minimized therapeutic efficacy, and hypersensitivity [92]. One strategy to overcome the drawbacks of PEGylation is the use of cleavable PEG derivatives. These environmentally sensitive derivatives can readily break under physiological and pathological conditions, detaching from the NP’s surface to promote its uptake by cells at the targeted site [93]. More research is still needed to evaluate the usability of these new derivatives.

### 6.3. PEG Alternatives

Owing to the limitations of the PEGylation strategy, many researchers have focused on the development of alternatives that can exhibit physicochemical properties similar to those exhibited by PEG without compromising the pharmacokinetic and therapeutic activities of the nanosystem [92]. Examples of PEG alternatives suggested for the coating of PLGA NPs include poly(2-oxazoline)s (POXs), CS, glycosaminoglycans, and poly(acrylamide)s.

POXs are thermosensitive, hydrophilic, biocompatible, and biodegradable polymers with better renal clearance than PEG [94]. They have been found to provide stealth effects and an acceptable ability to permeate mucosal tissues [95]. A library of poly(2-ethyl-2-oxazoline)-*b*-PLGA block copolymers with molar masses ranging from 6500 to 60,000 g·mol^−1^ has been synthesized. It was demonstrated that NPs formulated using these copolymers featured similar properties, such as surface electrical charge and hydrodynamic diameter, to those exhibited by PEG-PLGA-based NPs [96]. However, POXs are not yet FDA-approved and their synthesis is considered relatively difficult and costly, which may hinder their clinical application [92].

CS, a natural cationic polysaccharide produced through the partial deacetylation of chitin, is another polymer that is commonly used to coat the surface of PLGA NPs. It is biocompatible and biodegradable, and its –NH_2_ groups can be protonated at low pH, imparting a positive charge and, thus, mucosal adhesive properties to the macromolecule [97]. Therefore, CS-coated PLGA nanosystems can increase biocompatibility, protect from opsonization and improve retention and cellular uptake [98,99]. Additionally, CS coating has been used as a strategy to achieve sustained and pH-responsive drug release from PLGA nanostructures [100,101,102].

Heparin is a biocompatible sulfated glycosaminoglycan that shows a high binding affinity for various growth factors and has been used for the surface modification of delivery systems [103]. A study of the effects of heparin on PLGA NPs concluded that heparin functionalization resulted in a stable surface coating that increased in vitro cellular uptake and enhanced the accumulation of NPs in tumor-bearing mice by twofold more than the control. However, heparin was not effective at reducing accumulation in the liver in comparison with CS-modified PLGA NPs, which showed lower liver accumulation than the control [104]. Another member of the glycosaminoglycan family that has been applied to shield the surface of PLGA NPs is hyaluronic acid. This endogenous polymer provides a further important function, being a ligand of CD44 receptor, which can be exploited to target the membranes of some tumor cells that overexpress this receptor [105,106].

Poly(acrylamide)s are hydrophilic polymers with good biocompatibility and wide applications in the biomedical field. Among these synthetic polymers, poly(*N*-isopropylacrylamide) has been utilized to create a hydrophilic shell around PLGA NPs with the aim of providing protection from aggregation during lyophilization as well as providing temperature-responsive drug release [107]. Similarly, NPs based on a blend of poly(*N*-isopropylacrylamide-acrylamide-vinilpyrrolidone) with PLGA containing naltrexone and prepared using the emulsion–solvent evaporation method, showed sustained temperature-responsive drug release [108]. However, studies on the toxicity of and the immune response to the systemic use of these polymers are still required, especially given that acrylamide is known to be highly toxic [92].

### 6.4. Lipids

Phospholipids are used to modify the surface of polymeric NPs. Thanks to their amphiphilic nature, phospholipids can self-assemble in an aqueous medium. Lipid self-assembly on PLGA NPs is governed by electrostatic attraction and hydrophobic interactions between the lipids and the NP surface [109]. Thus, electrostatic attraction facilitates the adsorption of charged lipids in the form of bilayer vesicles onto the oppositely charged NP surface. Neutral phospholipids, however, can be adsorbed onto the hydrophobic surface of NPs through hydrophobic interactions. As a result, a lipid-monolayer surface coat is created in which the hydrophobic tails of the lipids are adsorbed onto the PLGA surface, whereas the lipids’ hydrophilic head groups extend into the external aqueous medium [109,110]. Among the lipids used for the surface modification of PLGA NPs, synthetic lipids, such as 1,2-dioleoyl-3-(trimethylammonium) propane (DOTAP), offer the advantage of ease of processing and customization. For example, the surface modification of PLGA NPs with the positively charged DOTAP enable them to adhere to surfaces of Gram-positive and Gram-negative bacterial pathogens, and provide sustained antibiotic delivery [111]. However, considering the possible increase in cytotoxicity and induction of immune response, alternative strategies, such as lipid vesicles derived from natural cell membranes (known as nanoghosts), have been investigated. Surface functionalization using cell membranes derived from cells, such as erythrocytes, leukocytes, platelets, stem cells, and cancer cells, equips the particle surface with unique cell mimetic features [112]. For example, purified erythrocyte nanoghosts fused around the PLGA surface through the extrusion method have been found to extend the blood circulation of NPs, provide better control over drug release, and enhance therapeutic efficacy in acute myeloid leukemia cells, compared with PEGylated PLGA NPs [113,114]. Furthermore, platelet membrane-surface-engineered PLGA NPs demonstrated natural platelet-mimicking functions, such as selective adhesion to damaged vasculatures and improved binding to platelet-adhering pathogens [115].

Overall, surface modification with lipids offers various advantages, including biomimetic and biodegradable properties, extended blood circulation, controlled drug release kinetics, the possibility of further surface modification and, consequently, improved therapeutic efficacy.

### 6.5. Surface Functionalization with Targeting Ligands

The surface functionalization of a nanosystem with a cell-targeting ligand that features the ability to recognize and bind to a particular biological target allows the delivery of encapsulated cargo to a specific cell population. Using this strategy, diseased cells, as in tumor cells, that overexpress a cell surface receptor that is poorly or not expressed on normal cells, can be actively targeted. Following ligand–receptor binding, NPs are internalized by receptor-mediated endocytosis.

**Figure 5:**
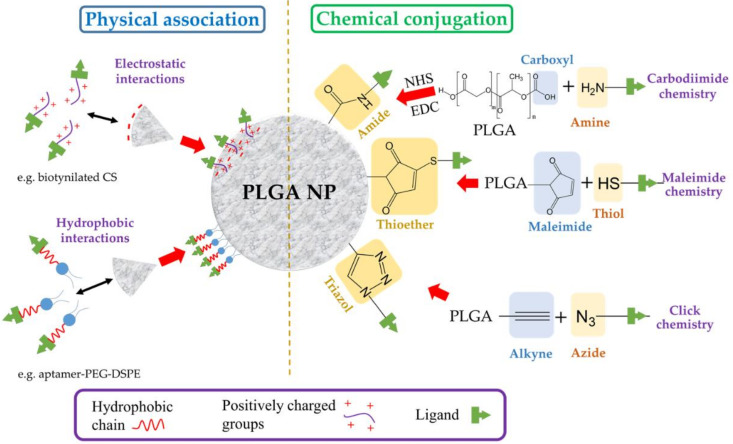
Major methods used for the attachment of targeting ligands on the surface of PLGA NPs. DSPE: 1,2-distearoyl-*sn*-glycero-3-phosphoethanolamine; EDC: 1-ethyl-3-(3-dimethylaminopropyl) carbodiimide; NHS: *N*-hydroxysuccinimide.

Both physical association, which involves simple electrostatic or hydrophobic interactions, or chemical covalent linking can be employed to attach the desired ligand on the surface of the particle (Figure 5). Additionally, systems with specific bindings, such as the avidin–biotin system, have also been used. In this approach, either biotin or avidin is first conjugated to the surface of the PLGA NP followed by the non-covalent linking of avidin- or biotin–ligand conjugates, respectively [116].

The chemical covalent conjugation of a targeting ligand to a NP is most frequently performed using carbodiimide chemistry to create an amide group between a primary amine group of the ligand and the terminal carboxyl group in the PLGA chain [117]. Briefly, a carbodiimide (such as 1-ethyl-3-(3-dimethylaminopropyl) carbodiimide (EDC)) reacts with the carboxylic acid groups of PLGA to form an amine-reactive *O*-acylisourea intermediate, which is derivatized into an *N*-hydroxysuccinimide (NHS) ester to prevent unwanted reactions. The formed intermediate enables the conjugation of a functional amino group of the ligand in the reaction mixture. Other conjugation strategies include the use of maleimide and click chemistry. The former maleimide groups of the NP are reacted with thiol moieties of the ligand, forming a thioether linkage [118], whereas in the latter, a reaction between azide and alkyne groups is involved [117]. It is very common to decorate the surface of the PLGA NP with PEG chains carrying terminal functional groups. This strategy brings about a double benefit: the stealth effect and the easy attachment of targeting ligands. A wide variety of ligands has been employed to equip the surface of PLGA NPs with active targeting (Table 5). The following sections are dedicated to providing a detailed discussion of these targeting ligands.

#### 6.5.1. Antibodies

Antibodies are among the most common ligands used for active targeting. They are able to specifically interact with surface antigens, thus generating improved therapeutic outcomes. Some recent examples of monoclonal antibodies (mAb) that have been conjugated to PLGA particles for the active targeting of tumors include anti-CD133 to target gastric carcinoma cells [119], anti-CD44 to target prostate cancer cells [120], anti-epidermal growth factor receptor (EGFR) to target breast cancer cells [121], cetuximab to target lung cancer cells [122], anti-PD-1 (Programmed cell death protein 1) antibodies to target CD8+ T cells [123], anti-PSMA (prostate-specific membrane antigen) to target prostate cancer cells [124], and trastuzumab (anti-human epidermal growth factor receptor 2 (HER2)) to target ovarian cancer cells [125] and human breast cancer cells [126,127]. In one of these studies, PLGA-PEG NPs encapsulating toremifene, with an average size of ≈250 nm, were developed and surface-decorated with anti-PSMA. After a three-week-treatment of nude male mice with orthotopic prostate tumors, the surface-functionalized particles significantly reduced the tumor size and the toremifene concentration in the tumor was 15 fold greater than in animals treated with the free drug [124]. In another interesting report, a PLGA-based NP was designed for the delivery of immunotherapies to the tumor by targeting specific subsets of endogenous immune cells. In this study, anti-PD1 antibodies were conjugated to PLGA NPs loaded with SD-208, a TGF*β*R1 inhibitor. It was hypothesized that following systemic administration, targeted PLGA NPs would bind to activated CD8+ T cells in the blood circulation and would be carried by these cells into the immunosuppressive tumor microenvironment, where the drug was released to restore the function of suppressed immune cells. Specific and efficient binding was confirmed in the in vitro and in vivo experiments, and delayed tumor growth and extended survival of tumor-bearing mice was observed only with targeted NPs, whereas drug-free and untargeted NPs showed no effect [123]. This approach opens up an exciting opportunity for the indirect targeting of cancer immunotherapy cells, and may be worthy of additional investigation to assess the clinical response in human subjects.

PLGA-based immunonanocarriers surface-functionalized with an antibody are normally engineered with a mean diameter in the range of 150 to 250 nm and a close-to-zero surface electrical charge (*ζ* ≈ 0 mV, due to the positively charged antibodies that neutralize the surface of the particle [4]. For the conjugation of mAb to the particle’s surface, covalent linking can be formed through carbodiimide [119,122,125], maleimide [120,121,123,124] or click [126] chemistry. Additionally, the positive charge of the antibody macromolecules can be exploited to provide physical electrostatic adhesion to the surface of the negatively charged particle [127].

Interestingly, antibody fragments can also be used to target the site of interest. For instance, single-chain antibody fragments (scFvs) of anti-CD133 were used to prepare tumor-targeting PEG-PLGA NPs [119]. scFvs are devoid of constant domains of antibodies that still feature the ability to bind to their specific antigens. Owing to their small size, scFvs can provide a greater cell penetration rate, as well as reduced immunogenic response in vivo [128]. In this scenario, PEGylated PLGA NPs loaded with pentamidine were surface-functionalized with single-domain, heavy-chain antibody fragments (nanobodies) that specifically recognized the surface of *Trypanosoma brucei*. It was demonstrated in vivo that this nanoformulation was capable of curing all infected mice in a murine model of African trypanosomiasis at a tenfold lower dose than the minimal full curative dose of the free drug [129].

#### 6.5.2. Biotin

Biotin (vitamin B7 or vitamin H), a water-soluble vitamin, is an essential promoter of cell growth and development. Biotin-selective transporters are overexpressed in some cancerous cells, whereas they show a limited presence in healthy tissues. In addition, biotin, as a small-molecule moiety, provides several advantages over large-molecule ligands, particularly its low or non-effect on the pharmacokinetics and immunogenicity of the nanosystem [130]. Therefore, biotinylated PLGA particles can be exploited as a promising platform for targeted drug delivery. For instance, several studies have shown an elevation in anticancer uptake by cells treated with biotin-targeted PLGA NPs compared to those incubated with a non-targeted particle or free drug [131,132]. Obviously, this is associated with an increase in the drug cytotoxicity observed by a significant reduction in cancer cell viability. Furthermore, the superiority of biotinylated PLGA NPs over non-targeted NPs has also been confirmed in vivo [133].

The targeting moiety is usually coupled to the polymer chain before the fabrication of the NP. For instance, the biotin moiety can be covalently linked to PEG-bis-amine, and the resulting PEG-biotin can be conjugated to PLGA through a carbodiimide coupling reaction. Next, the targeted NPs, characterized by a mean size and ζ of ≈180 nm and ≈−5 mV, respectively, are prepared by the emulsification solvent evaporation method [131].

In addition, biotin-decorated PLGA NPs have been suggested for targeted delivery to the posterior segment of the eye. This suggestion was based on the fact that the sodium-dependent multivitamin transporter (SMVT) that selectively uptakes biotin through active transport is expressed by the retinal pigment epithelium. Lutein-loaded PLGA-PEG-biotin NPs fabricated by o/w solvent evaporation, with a size of ≈208 nm and *ζ* of ≈−27 mV, demonstrated a higher drug uptake compared to non-targeted NPs and lutein alone [134]. However, efficacy, safety, and pharmacokinetics studies in preclinical models are necessary to confirm these preliminary findings and the usability of this system in the treatment of age-related macular degeneration.

#### 6.5.3. Bisphosphonate

Bisphosphonates (diphosphonates), or BPs, which can be divided into aminobisphosphonates (e.g., alendronate, ibendronate, pamidronate, zoledronate) and non-aminobisphosphonates (e.g., clodronate, etidronate), are highly effective inhibitors of bone resorption that suppress osteoclast function by inducing apoptosis. They are clinically indicated for the prevention of bone mass loss and the treatment of excessive bone resorption disorders, such as osteoporosis and bone metastasis [135]. The structure of BPs is characterized by two phosphonate groups sharing a common carbon atom (P-C-P) that demonstrate a high affinity for calcium phosphate surfaces, and thus features the ability to selectively target bone minerals. It is therefore hypothesized that BP surfaces linked to PLGA NPs not only enable targeted delivery to the bones but can also contribute therapeutically by preventing tumor-associated bone resorption.

BPs with primary amine groups, such as alendronate [136,137,138], or an acidic carboxylate group, such as zoledronic acid [139,140], can be linked to PLGA, or its PEG copolymer, with end carboxyl or amine groups. Such BP-surface-decorated PLGA NPs have presented satisfying bone targeting effects in vitro and in vivo. For example, a recent study developed zoledronic acid-decorated PLGA NPs loaded with multiple anticancer drugs namely, gemcitabine and epirubicin. In vitro studies showed that the targeted NPs exhibit a good ability to bind to human bone particles and increase cellular uptake in osteosarcoma cells (MG-63 cell line) in comparison with free drugs. In addition, in vivo antitumor studies carried out over four weeks on rats confirmed the enhanced efficacy of the targeted NPs, which was demonstrated by a considerable reduction in tumor volume, which was ≈250% lower in rats treated with zoledronic acid-functionalized NPs compared to PBS-treated rats [140].

Surface modification with BPs may also be used to attain controlled drug release. In this context, a nanosystem comprising hydroxyapatite molecules, the major inorganic component of bone and teeth in mammals, linked to the surface of pamidronate-modified PLGA NPs was suggested. Pamidronate was first conjugated with the non-ionic surfactant Brij^®^ 78 (polyoxyethylene 20 stearyl ether) and the conjugate was then used in the fabrication of the NPs. The high affinity of surface pamidronate moieties was exploited to form a conjugate with hydroxyapatite, which can be used to construct drug-releasing, hydroxyapatite-based bone graft substitutes [141].

#### 6.5.4. Folate

Folic acid (FA), vitamin B9, is a water-soluble small molecule that is stable over a broad range of temperatures and pHs, inexpensive, non-immunogenic, and essential for the biosynthesis of nucleotides and cell division. The folate receptor, a cell surface receptor anchored to the plasma membrane by glycosylphosphatidylinositol, is highly overexpressed in a wide range of cancer lesions, such as brain, breast, cervical, colorectal, epithelial, kidney, lung, and ovarian tumors. However, in healthy tissues, the expression of these receptors is restricted to the kidneys, lungs, choroid plexus, and placenta [142]. Furthermore, low-folate diets are associated with elevated risk of some tumors [143].

Folate receptors show a high affinity for FA, which is maintained even when FA is conjugated to other compounds, allowing FA-modified NPs to target tumors that express folate receptors. After internalization through endocytosis, FA is dissociated from its receptor as the pH in the endosome approaches five, liberating the drug-entrapped particle [142]. These NPs are usually produced by the covalent linking of FA to a conjugate of PLGA with PEG with an amine end group through carbodiimide chemistry [14].

Several recent studies have re-confirmed the considerable ability of FA-decorated PLGA NPs to promote the targeted delivery and cellular internalization of anticancer agents by different tumor cells [14,144,145,146]. For example, loading 5-FU into FA-PEG-PLGA NPs resulted in almost fourfold lower IC_50_ (half-maximal inhibitory concentration) compared with 5-FU-loaded PLGA NPs, as observed by the cytotoxicity study in folate-overexpressed HT-29 colon cancer cells and MCF-7 breast cancer cells [14].

A number of researchers have investigated the biodistribution of FA-PEG-modified PLGA NPs. FA-modified NPs labelled with indocyanine green (ICG), a near-infrared fluorescence dye, administered intravenously to mice xenografted with MDA-MB-231 human breast cancer cells, showed a significant accumulation in tumor compared to non-modified particles. In comparison with non-modified NPs, an increase in the area under the curve (AUC, from 0 to 12 h) in plasma and in tumors by 245% and 194%, respectively, and a reduction in the AUC (from 0 to 12 h) in the liver by 13% by targeted stealth NPs was reported [147]. Likewise, using a similar FA-ICG-PLGA nanosystem, it was shown that NPs injected subcutaneously into mice with MCF-7 tumors exhibited much greater circulation times than free ICG and specifically targeted tumors [148]. Furthermore, PEG-PLGA NP surfaces modified by FA and K237 (a polypeptide that targets the vascular endothelial growth factor receptor-2) and radiolabeled with ^99m^Tc, a radionuclide nuclear agent, were found to accumulate in tumors, the liver, kidney, spleen, and blood at 3 and 9 h after intravenous administration in mice xenografted with ovarian cancer cells (SKOV-3 cells) [149].

#### 6.5.5. Lectins

Lectins are proteins that recognize and bind to carbohydrate complexes linked to lipids and proteins. They show high specificity for glycosylated cell-surface components. Different glycan arrays are expressed by different cells. Thus, lectins can be employed in the selective targeting of unhealthy cells, such as cancer cells, that frequently express different glycans from those present in their normal counterparts [150]. One example is wheat germ agglutinin (WGA), a 36-kDa lectin present in *Triticum vulgare*, which features four binding sites specific for *N*-acetylglucosamine and sialic acid (SA) residues on the cell membrane. These sugars are ubiquitously present in tumors, the intestine, and the nasal cavity, and upon binding, WGA is rapidly internalized via receptor-mediated endocytosis.

It was shown that the transcellular transport of WGA-NP in Caco-2 cells mainly takes place through a clathrin-mediated mechanism. It was also found that WGA surface-decorated PEG-PLGA NPs, with a mean diameter of ≈120 nm, exhibited greater lysosome escape and improved transcellular transport compared with PLA NPs. Furthermore, the same study reported that NPs with shorter PEG surface chains (2 kDa) resulted in enhanced cellular association and higher cellular uptake compared to NPs conjugated with larger PEG chains (5 kDa) [151].

Several studies have demonstrated that PLGA NPs decorated with lectins, such as dorranalectin [152,153] and *Solanum tuberosum* lectin [154], are potentially efficient at nose-to-brain targeted delivery. Furthermore, gastro-retentive NPs with anti-*Helicobacter pylori* activity were designed by using concanavalin-A (a lectin isolated from jack-bean (*Canavalia ensiformis*))-decorated PLGA NPs and loaded with acetohydroxamic acid and clarithromycin. The decorated NPs were found to enhance mucoadhesion, which was attributed to the lectin polysaccharide binding ability, and to provide sustained drug release over a period of 8 h in comparison with non-conjugated NPs [155].

#### 6.5.6. Mannan

Mannan (MN) is a biodegradable polysaccharide that is able to specifically bind to mannose receptors. The mannose receptor is a member of the C-type lectin receptor family and is mainly expressed on macrophages and dendritic cells, which can thus be targeted using MN as a ligand [156].

PLGA NPs decorated with MN have been proposed as gene delivery vehicles to target Kupffer cells (KCs) in the liver. For example, MN was conjugated to L-*α*-phosphatidylethanolamine (PE) and the MN–PE conjugate was used to coat the previously prepared particles through physical adhesion to the surface. This procedure led to the formation of NPs with a mean size of 190 nm and a minimized *ζ* of −15.46 mV, owing to the effect of the MN-PE coat. Loaded with plasmid-enhanced green fluorescent protein, the intravenously administered MAN-NPs demonstrated greater transfection efficiency (39%) in comparison with non-targeted PLGA NPs (25%) and the commercial liposome (23%) in liver KCs, measured 48 h post-injection in rats [157]. Similar findings were obtained when a PEG chain (2 kDa) was incorporated into the NP coatings. Thus, MN-PEG-PE-coated PLGA-DNA NPs resulted in considerably enhanced transfection efficiency in rat liver KCs up to 72 h post-intravenous administration due to the extended circulation time [156].

In addition, MN-decorated PLGA NPs have demonstrated immunoadjuvant activity, as evidenced by the enhanced stimulation of dendritic cell phenotypic and functional maturation [158]. Furthermore, when MN-PLGA NPs carrying a model antigen (ovalbumin) were examined in vitro and in vivo (in mice), they were found to considerably boost antigen-specific CD4+ and CD8+ T-cell responses in comparison with untargeted NPs [159]. Therefore, these systems may be useful for designing more efficient vaccine formulations based on PLGA NPs.

#### 6.5.7. Nucleotides

Aptamers (Aps) are short, single-stranded DNA or RNA able to form unique tertiary conformations that selectively bind to specific target antigens, such proteins, peptides, and carbohydrates with high affinity, analogous to antibodies. Aps offer advantages over antibodies insofar as they are much more versatile in synthesis and modification; they offer exhibit various benefits, such as their high stability and small size, which make them able to easily penetrate solid tumors, as well as their low immunogenicity, low fabrication cost, and scalability [160].

Similar to antibodies, oligonucleotide Aps can be designed against any cancer-related biomarker. The attachment of these Aps to a nanoassembly has been the focus of recent investigations on cancer chemotherapy [161]. As an example, an A15 Ap specific to CD133, a cancer stem cell (CSC) marker of osteosarcoma, was decorated on PLGA NPs (particle size ≈ 150 nm) for the targeted delivery of salinomycin, a polyether ionophore antibiotic with CSCs cytotoxic activity, to osteosarcoma CSCs. In a cell proliferation assay, Ap-decorated NPs showed 4.92 fold higher therapeutic efficiency compared to non-targeted NPs. Furthermore, preclinical studies on animals reported a threefold decrease in tumor size compared to the control, with no considerable weight loss [162]. In addition, an RNA Ap specific to Ets1, an oncogenic transcription factor, conjugated to PLGA particles, was shown to increase gefitinib’s anticancer effects specifically in Ets1-overexpressing, gefitinib-resistant H1975 lung cancer cells. Furthermore, a considerable reduction in tumor growth in H1975 xenograft nude mice was reported after a single intratumoral injection of the Ap bioconjugate [163].

Brain-penetrating Aps that specifically bind to biomarkers overexpressed on the targeting tumor have also been synthesized. For example, a Gint4.T Ap, specific for PDGFR*β* (platelet-derived growth factor receptor beta) overexpressed on the glioblastoma cell line, U87MG, was integrated with PEG-PLGA NPs. The Ap-decorated NPs encapsulated with the lipophilic BODIPY dye (for visualization purposes) were rapidly internalized 10 min post-incubation, and considerably increased the fluorescence intensity of the whole brain and tumors of mice 2 h and 4 h post-administration, respectively. For therapeutic purposes, the NPs were loaded with dactolisib, a potent PI3K-mTOR inhibitor, and examined in the GBM orthotopic mice model. The anticancer efficacy was confirmed by the significantly reduced levels of phospho.4EBP1 in tumors harvested from the treated mice groups, in comparison with the controls [164].

These recent studies present the potential application of Apt-PLGA NPs to the inhibition of tumors and the prolongation of patient survival. 

#### 6.5.8. Peptides

Peptides have attracted a significant amount of interest as ligands due to their small size, low toxicity, lack of immunogenicity, high selectivity, and wide inter/intracellular [165]. Peptide-modified PLGA-based NPs have recently been shown to successfully target various diseases, such as cancer [166,167] and atherosclerotic plaques [118].

Among these, cell-penetrating peptides (CPPs) are emerging as effective tools to facilitate the translocation of nanosystems across biological membranes. Generally, CPPs are small-sized compounds composed of domains of 5 to 30 amino acids [168]. The cellular penetration of CPPs usually takes place in various pathways, divided mainly into energy-independent direct translocation or energy-dependent endocytosis. While direct penetration is observed when high concentrations of CPPs are used, endocytosis mainly occurs when the CPP is linked to larger cargos. It is also believed that electrostatic interaction between positively charged CPPs and negatively charged cellular membranes facilitates surface binding, which is consequently followed by internalization via distinct entry mechanism acids [168]. Investigations of cell penetration by CPP-modified PLGA NPs have confirmed the involvement of the endocytic entry mechanism [169,170,171]. The penetration efficiency of various CPPs linked to PLGA NPs was recently explored. The non-covalent attachment of four CPPs, including low M_W_ protamine (LMWP), penetratin, Tat, and poly(arginine) 8, was attained through the electrostatic forces between the positively charged CPPs and the negatively charged surface of the particle. Among the studied CPPs, LMWP provided enhanced cellular uptake and improved partition of NPs into cochlear tissues after administration to guinea pigs [172]. Furthermore, PLGA-based NPs were double-functionalized with CCP (either R8, Tat, or penetratin) and a secretion peptide (Sec), a 16 amino acid sequence derived from the second and third helix of the engrailed homeodomain, to enhance transport across the intestines. It was hypothesized that while CCPs facilitate the internalization step, Sec can induce secretion outside the intestinal epithelium cells, thus promoting transcellular transport. Among the examined NPs, Sec/penetratin NPs significantly increased transcellular transport across Caco-2 cells and increased the bioavailability of insulin upon ileal segment administration in rats [173]. However, just as CPPs in general are non-selective and penetrate almost all cells, likewise, the further surface functionalization of NPs with a targeting ligand may be required for efficient delivery to the site of action.

BBB-penetrating peptides, such as angiopep-2 peptide [174] and DWSW peptide [166], are exciting tools with which to target brain diseases. Preclinical studies of the systematic administration of these PLGA-based systems showed the ability of BBB-penetrating peptides to facilitate the entry of PLGA into the brain [166].

The RGD peptide family features a specific affinity with integrin receptors, which are heterodimeric transmembrane subunits of *α* and *β*. These receptors are highly expressed in specific healthy tissues and in various diseased tissues, making them useful targets for NP-based diagnosis and treatment strategies [175]. For example, RGD peptide-functionalized PLGA NPs have shown promise in targeting drug delivery to the endothelial cells of tumor vasculature that overexpress *α*_v_*β*_3_ integrin [176]. Using PEGylated PLGA NPs, the effects of two different RGD peptides were characterized: one is cyclic (RGDFC), and is known to bind specific affinity with *α*_v_*β*_3_ integrin heterodimers; and the other is linear (RGDSP), and features equal affinity with *α*_v_*β*_3_ and *α*_5_*β*_1_. The study showed that particles with RGDFC were greatly taken up by glioma U87MG (which is very high in *α*_v_*β*_3_ and moderate/high in *α*_5_*β*_1_) but not by ovarian carcinoma A2780 (which features almost no *α*_v_*β*_3_ and is moderate in *α*_5_*β*_1_). RGDSP demonstrated similar internalization in both cell lines and no more than 50% of the uptake shown by the cyclic peptide, which may be related to its possible low and non-specific affinity with different integrins. Thus, these findings show the possibility of targeting cells with specific integrins, using various RGD peptides [177].

Another strategy to enhance the cellular uptake of nanocarriers is functionalization with peptides that specifically bind to the intracellular cell adhesion molecule-1 (ICAM-1), a cell surface glycoprotein that is overexpressed in various pathologies, such as inflammation [178]. In this context, cyclo(1,12)PenITDGEATDSGC (cLABL) peptide-functionalized PLGA NPs were shown to effectively bind to ICAM-1 on lung epithelial cells followed by receptor-mediated cellular internalization [179].

#### 6.5.9. Sialic Aid

SAs, acidic nine-carbon monosaccharide derivatives of neuraminic acid, are components of various cell surface glycoproteins and glycolipids and play a vital role in many physiological and pathological processes. SA receptors are divided into three types: selectins, factor H protein, and siglecs (SA-binding immunoglobulin-like lectins) [180]. Selectin, a transmembrane glycoprotein, contributes to tumor metastasis through the modulation of cell–cell interactions among cancer, leukocytes, platelets, and endothelial cells [181]. In addition, selectin is also overexpressed on endothelial cells during inflammation. Taking this phenomenon into account, PLGA-based NPs were modified by SA and loaded with lycopene, a naturally occurring antioxidant, to target inflamed endothelial cells. The suitability of this nanosystem was evidenced by the significant reduction in pro-inflammatory cytokine levels, which seems to have been attributed to the enhanced internalization of the NPs [182].

Furthermore, siglecs are a family of type I transmembrane proteins containing an SA-binding site at *N*-terminus. Among siglec receptors, CD22, which highly expressed in B-cell derived non-Hodgkin’s lymphoma, has been identified as a target for effective anticancer treatment by decorating a PLGA-based nanosystem with the SA moiety [183].

Among different organs, the brain shows a relatively high expression of SA receptors, making it a suitable target for active drug delivery. This was demonstrated by the enhanced brain accumulation of SA-decorated PLGA NP and additionally functionalized with similopioid, a BBB-penetrating peptide. One major drawback of this strategy is the presence of SA receptors in other organs, which leads to undesirable drug accumulation in those organs [184].

On the other hand, endogenous SA can itself be exploited as a target for drug delivery. For example, PLGA NPs with a positive surface charge owing to the surface adsorption of the anticancer drug, doxorubicin (positively charged at physiological pH), was shown to interact electrostatically and consequently to bind to SA (negatively charged at physiological pH), thus facilitating cellular uptake [185].

#### 6.5.10. Transferrin

Transferrin (Tf) is a glycoprotein, a hydrophilic transport vector (composed of 700 amino acids with a M_W_ of 80 kDa) that is generated within the brain and liver and features the ability to bind iron and to control its levels in body fluids. Tf receptors, which facilitate the cellular internalization of iron though interaction with Tf, are present on both normal cells (such as erythrocytes, hepatocytes, intestinal cells, and epithelial cells of BBB) and cancer cells. However, owing to the high rate of proliferation and the extensive need of iron, Tf receptors are much more overexpressed on the surface of various tumor types, and their expression can reach up to 100 fold that encountered in normal cells [186]. In line with this, it has been shown that the surface decoration of PLGA NPs with Tf ligands remarkably enhances their the efficiency with which they target various tumors with high expression of Tf receptors, as observed in breast cancer [187,188], pancreatic cancer [189], lungs cancer [190,191], and brain glioma [187,192].

Additionally, Tf receptors are highly present on the endothelial cells of the BBB; hence, targeting these receptors creates an exciting opportunity for drug delivery to the brain. In this context, PLGA NPs with a particle size between 80 and 90 nm surface-modified with either Tf or bovine serum albumin (BSA), were designed and their behavior in biological systems was compared. In the in vitro studies, Tf-NP internalization by F98 glioma cells was significantly higher compared with that of BSA-NPs, supporting specific interaction between Tf and overexpressed Tf receptors. Upon intravenous administration in mouse and rat models, both Tf- and BSA-modified PLGA significantly prolonged blood half-life compared with blank NPs. The in vivo study on brain targeting showed that Tf-NPs can penetrate across healthy BBB at higher concentrations compared to BSA-NPs, although both NPs demonstrated similar targeting patterns in F98 tumor-bearing rats [193]. With the aim of overcoming the poor transport across the BBB and the low penetration across the blood–tumor barrier, Tf-conjugated magnetic silica PLGA NPs loaded with both doxorubicin and paclitaxel were designed for the treatment of brain glioma. In the in vitro experiments, improved cytotoxicity and cellular uptake efficiency were observed with the Tf-conjugated PLGA NPs loaded with the two anticancer agents. These findings were in line with in vivo results in which the Tf- and magnetic-targeted NPs demonstrated enhanced antiglioma efficacy in intracranial U-87 MG-luc2 xenograft of BALB/c nude mice [194]. Similar findings were also observed using Tf-PLGA NPs loaded with doxorubicin. It is noteworthy that the functionalization of PLGA NPs with moieties that feature a specific affinity with Tf receptors, such as peptides [85] and antibodies [195], have also confirmed the importance of Tf receptor-mediated uptake at the BBB as an efficient strategy to target the brain.

## 7. Conclusions

Owing to their versatility and unique properties, PLGA NPs represent an effective platform with exciting opportunities in the delivery of therapeutics. Surface functionalization, such as PEGylation and ligand-surface decoration, are effective strategies in equipping PLGA nanocarriers with desired functions for effective performance in biological systems, such as increased systemic circulation, enhanced uptake by biological membranes, and specific cellular and intercellular targeting abilities. A plethora of materials for coating PLGA NPs has already been investigated and proven to be beneficial for optimizing NP properties. Selection criteria for suitable coating materials should take into consideration the desired application, such as using a CS coat for PLGA NPs intended for genetic or mucosal delivery. Thanks to the development of simple and effective functionalization methods, the majority of targeted PLGA NPs described in previous research use the double surface functionalization strategy, in which a hydrophilic coat and a targeting ligand are combined in the PLGA-based nanosystem. This strategy has been proven to be effective at prolonging the biological half-life of PLGA NPs, as well as achieving active targeting and facilitating cellular uptake, as evidenced by in vivo findings.

The outcomes of current preclinical research on PLGA nanocarriers are promising. However, many of the conducted studies lack a critical reflection on the clinical feasibility of the used approaches. The design of an ideal PLGA-based nanomedicine that better matches its biomedical requirements and that can advance from bench to bedside still faces several challenges. While surface coating with hydrophilic polymers to produce stealth NPs may prevent their recognition by the immune system, it has been reported to increase particle size, hide targeting moieties, and reduce cellular internalization. Furthermore, any minor chemical modification to the surface of the particle, as well as to the formulation, may alter its physicochemical features, which in return influences both the in vitro and the in vivo performance, as well as the therapeutic efficacy of the PLGA-based nanostructure. Therefore, particle surface modification should always be associated with intensive characterization of the modified nanosystem in terms of toxicity, biocompatibility, stability, pharmacokinetics, on-target and off-target biodistribution and accumulation, and pharmacodynamics. A potentially successful approach should, from the beginning, tailor the experimental setup based on a specific clinical problem as well as considering other fabrication challenges, such as batch-to-batch reproducibility, pharmaceutical upscaling, quality control, good manufacturing practice requirements, and economical production.

## Figures and Tables

**Table 1:** The influence of PLGA composition on the polymer’s physical properties.

Property	Effect	Polymer Composition
**Hydrophilicity**	↑	↓ lactide content ↓ M_W_
**Glass transition temperature (Tg)**	↓	↓ lactide content ↓ M_W_
**Crystallinity**	Amorphous	Poly(glycolide) < 70% Lactide segment: poly(d,l-lactide)
	Crystalline	Lactide segment: poly(l-lactide)

**Table 2:** Major methods used in the fabrication of PLGA NPs.

Method	Procedure	Size Range (nm)	Advantages	Disadvantages	Examples
**Emulsion evaporation**	A non-water miscible solvent containing PLGA is emulsified with an aqueous solution containing a surfactant using high shear force	≈50 to 700	Relatively non-toxic, small particle size, easy to scale up, and can be used to encapsulate both water-soluble and water-insoluble drugs	Drug stability may be affected during high energy mixing, and long solvent removal step	[27,28,29,30]
**Nanoprecipitation**	A water-miscible solvent containing PLGA is dispersed into an aqueous phase using low energy mixing	≈80 to 700	Simple, rapid, narrow size distribution, and non-toxic solvents and low energy are used	Low entrapment efficiency of polar drugs, long solvent removal step, and particle size is considerably affected by polymer concentration	[31,32,33]
**Solvent diffusion**	A partially water miscible solvent containing PLGA is emulsified with an aqueous solution of a suitable surfactant	≈50 to 400	Toxic solvents and high stress shear are avoided	Large quantities of water and long agitation time are required, polymer concentration notably affect the particle size, and low entrapment efficiency of polar drugs	[34,35,36]
**Salting out**	A water-miscible solvent containing PLGA is emulsified with an aqueous phase containing a high concentration of salts under high shear stress agitation. The resulting o/w emulsion is diluted with water	≈100 to 500	Rapid, high concentrations of PLGA can be used, no high stress shear is required, and suitable for heat-sensitive drugs	Purification step is needed, solvents used may be explosive, and not suitable for lipophilic drugs	[37,38,39,40]

**Table 3:** Overview of materials used for coating the PLGA particles.

Coating Material	Examples	Advantages	Disadvantages
Surfactants	PVA, poloxamers, polysorbates, sodium cholate, vitamin E TPGS	Preventing NP aggregation, reduced size and uniform distribution, and sustained drug release	Potential toxicity
PEG	–	Stealth effect, prolonged blood circulation time, and enhanced mucus penetration	Compromised drug activity, non-biodegradability with potential accumulation in the body, and potential immunogenicity
PEG alternatives	Poly(2-oxazoline)s, glycosaminoglycan, poly(acrylamide)s, and CS	Biodegradable, stealth effect, mucosal adhesiveness (CS), improved cellular uptake, and sustained drug release	Poor solubility (CS), cost, and potential toxicity
Phospholipids	Erythrocyte, platelet membranes, nanoghosts, and 1,2-dioleoyl-3-(trimethylammonium) propane (DOTAP)	Biomimetic and biodegradable properties, extended blood circulation, and controlled drug release	Increased cytotoxicity, and induction of immune response

**Table 4:** PLGA-based NPs that have undergone clinical trials in the last decade.

Name/Company	Surface Functionality	Drug	Investigated Application	ClinicalTrials.gov Identifier Number/Status
BIND-014/BIND Therapeutics	PEG	Docetaxel	Advanced urothelial carcinoma, cervical cancer, cholangiocarcinoma or carcinomas of the biliary tree and squamous cell carcinoma of the head and neck	NCT02479178/Phase II completed (January 2020)
			v-Ki-ras2 Kirsten rat sarcoma viral oncogene homolog (KRAS) mutation positive or squamous cell non-small cell lung cancer (NSCLC) that have progressed after treatment of one prior platinum-containing chemotherapy regimen	NCT02283320/Phase II completed (April 2016)
			Metastatic castration-resistant prostate cancer	NCT01812746/Phase II completed (April 2016)
			Advanced NSCLC	NCT01792479/Phase II completed (April 2016)
			Advanced or metastatic cancer	NCT01300533/Phase I completed (February 2016)
RECIOUS-01/Radboud University	–	IMM60 and NY-ESO-1	Advanced solid tumor (immunomodulatory)	NCT04751786/Phase I recruiting (Estimated study completion date: December 2022)

**Table 5:** Summary of recently developed PLGA-based NP surfaces functionalized with targeting ligands. 5-FU: 5-fluorouracil; CPP: cell-penetrating peptide; DSPE-PEG: 1,2-distearoyl-*sn*-glycero-3-phosphoethanolamine-*N*-[amino(polyethylene glycol)]; ICG: indocyanine green (fluorophore); PSMA: prostate-specific membrane antigen; RGD: arginine-glycine-aspartic acid.

Targeting Ligand	Ligand Attachment Technique	Preparation Method	Stabilizer	Other Surface Modifications	Mean Size (nm)	Therapeutic Agent	Application/Experiments Performed	References
**Antibodies**								
Anti-CD133	Carbodiimide chemistry	Double emulsion solvent evaporation	Tween^®^ 20/PVA	PEG	≈175	Methioninase/pemetrexed	Gastric carcinoma/In vitro	[119]
Anti-CD44	Maleimide chemistry	Emulsification solvent evaporation	PVA (30 to 70 kDa)	Lipid film (phosphatidylcholine, DSPE, cholesterol)/ PEG	≈140	Salinomycin	Prostate cancer cells/In vivo	[120]
Anti-EGFR protein	Maleimide chemistry	Nanoprecipitation	–	PEG	≈335	Paclitaxel	Breast cancer/In vivo	[121]
Cetuximab	Carbodiimide chemistry	Emulsification solvent evaporation	PVA (30 to 50 kDa)	–	≈130	Docetaxel	Lung cancer/In vitro and in vivo	[122]
Anti-PD-1	Carbodiimide chemistry	Emulsification solvent evaporation	PVA (30 to 70 kDa)	PEG	≈270	SD-208 (inhibitor of TGF-*β* kinase)	Immunotherapy—CD8+ T cells targeting/In vitro and in vivo	[123]
PSMA antibody	Maleimide chemistry	Emulsion solvent diffusion	PVA	PEG	≈250	Toremifene	Prostate cancer cells/In vitro and in vivo	[124]
Trastuzumab	Carbodiimide chemistry	Nanoprecipitation	PVA (9 to 10 kDa)	CS	≈125	Cisplatin	Ovarian cancer/In vitro	[125]
	Click chemistry	Nanoprecipitation	–	PEG	≈100	Doxorubicin	Breast cancer/In vitro	[126]
	Physical electrostatic adhesion	Emulsion solvent diffusion	PVA (13 to 23 kDa)	Polyethylenimine/phosphatidylcholine	≈220	Docetaxel	Breast cancer/In vitro	[127,128,129,130]
**Biotin**	Carbodiimide chemistry	Emulsification solvent evaporation	PVA (20 to 30 kDa)	PEG	≈180	SN-38	Breast cancer/In vitro	[131]
	Carbodiimide chemistry	Emulsification solvent evaporation	PVA	PEG	≈180	15,16-Dihydrotanshinone I	Cervical cancer/In vitro	[132]
	Carbodiimide chemistry	Nanoprecipitation	PVA (30 to 70 kDa)	CS	≈220	Epirubicin	Breast cancer/In vitro and in vivo	[133]
	Not mentioned	Emulsification solvent evaporation	PVA (30 to 70 kDa)	PEG	≈210	Lutein	Delivery to the posterior segment of the eye/In vitro	[134,135]
**Bisphosphonates**								
Alendronate	Carbodiimide chemistry	Nanoprecipitation	Pluronic^®^ F-68	–	≈200	N/A	Osteolytic bone metastases/In vitro	[136]
	Carbodiimide chemistry	Emulsification solvent evaporation	Pluronic^®^ F-68	–	≈245	doxorubicin	Bone cancer/In vitro and in vivo	[137]
	Physical adhesion	Emulsification solvent evaporation	PVA (30 to 70 kDa)	–	≈235	Curcumin/bortezomi	Bone cancer/In vitro and in vivo	[138]
Zoledronic acid	Carbodiimide chemistry	Nanoprecipitation	Pluronic^®^ F-68	PEG	≈130	Docetaxel	Bone cancer/In vitro and in vivo	[139]
	Carbodiimide chemistry	Nanoprecipitation	Pluronic^®^ F-68	–	≈190 to 245	Gemcitabine/epirubicin	Bone cancer/In vitro and in vivo	[140]
Pamidronate	Physical adhesion	Emulsification solvent evaporation	Brij^®^ 78	–	≈155	Curcumin	–	[141,142]
**Folic acid**	Carbodiimide chemistry	Nanoprecipitation	PVA	PEG	≈190	5-FU	Colon and breast cancer/In vitro	[143]
	Physical incorporation of DSPE-PEG-FA	Nanoprecipitation	–	Phospholipids/PEG	≈200	Pheophorbide	Gastric cancer/In vitro and in vivo	[144]
	Physical adhesion using a folic acid–dodecylamine conjugate	Emulsification solvent evaporation	PVA (30 to 70 kDa)	–	≈230	Docetaxel	Breast adenocarcinoma/In vitro and in vivo	[145]
	Carbodiimide chemistry	Emulsification solvent evaporation	PVA (13 to 23 kDa)	PEG	≈200	Oxaliplatin	Colorectal cancer/In vitro and in vivo	[146]
	Carbodiimide chemistry	Emulsion solvent diffusion	PVA	PEG	≈280	ICG	Breast cancer/In vivo	[147]
	Physical incorporation of DSPE-PEG-FA	Nanoprecipitation	–	Lecithin/DSPE-PEG	≈100	ICG	Tumor diagnosis and targeted imaging/In vitro and in vivo	[148]
	Carbodiimide chemistry	Emulsification solvent evaporation	Pluronic^®^ F-68	PEG/polypeptide K237	≈105 to 130	Technetium-99 (^99m^Tc, radiolabeled)	Ovarian cancer/In vitro and in vivo	[149]
**Lectins**								
Wheat germ agglutinin	Maleimide chemistry	Emulsification solvent evaporation	Sodium cholate	PEG	≈120 to 135	Curcumin	Enhanced transcellular transport/In vitro	[150,151]
Odorranalectin	Maleimide chemistry	Double emulsion solvent evaporation	Sodium cholate	PEG	≈115	Urocortin peptide	Nose-to-brain delivery-Parkinson’s disease/In vivo	[152,153]
Solanum tuberosum	Maleimide chemistry	Emulsification solvent evaporation	Sodium cholate	PEG	≈125	–	Nose-to-brain delivery/In vitro and in vivo	[154]
Concanavalin-A	Carbodiimide chemistry	Nanoprecipitation	Pluronic^®^ F-68	–	≈550 to 700	Clarithromycin/acetohydroxamic acid	Helicobacter pylori infection/In vitro and ex vivo bioadhesion	[155]
**Mannan**	Carbodiimide chemistry	Emulsion solvent diffusion	Carbopol^®^ 940	PEG-PE	≈215	Plasmide DNA	Targeted gene delivery/In vitro and in vivo	[156]
	Carbodiimide chemistry	Nanoprecipitation	Carbopol^®^ 940	PE	≈190	Plasmide DNA	Targeted gene delivery/In vitro and in vivo	[157]
	Physical adsorption or carbodiimide chemistry	Double emulsion solvent evaporation	PVA (30 to 50 kDa)	–	≈300 to 500	–	Vaccine formulations/In vitro	[158]
	Carbodiimide chemistry	Double emulsion solvent evaporation	PVA (30 to 50 kDa)	–	≈405	Ovalbumin	Antigen-specific T-cell responses/In vitro and in vivo	[159]
**Aptamers**								
Heparanase	Carbodiimide chemistry	Nanoprecipitation	TPGS	PEG	≈145	Paclitaxel	Breast cancer/In vitro and in vivo	[160,161]
CD133 aptamers	Carbodiimide chemistry	Emulsification solvent evaporation	Sodium cholate	PEG	≈150	Salinomycin	Osteosarcoma cancer stem cells targeting/In vitro and in vivo	[162]
RNA aptamer specific for Ets1	Carbodiimide chemistry	Emulsification solvent evaporation	PVA	–	Not specified	Gefitinib	Lung cancer/In vitro and in vivo	[163]
Gint4.T aptamer (anti-PDGFR*β*)	Carbodiimide chemistry	Double emulsion solvent evaporation	Sodium cholate	PEG	≈50	Dactolisib	Glioblastoma/In vitro and in vivo	[164]
**Peptides**								
S2P	Maleimide chemistry	Emulsification solvent evaporation	PVA (30 to 70 kDa)	PEG	≈185	Imatinib	Atherosclerotic plaques/None	[165]
^D^WSW and NGR	Maleimide chemistry	Nanoprecipitation	-	PEG/erythrocyte membranes	≈150	Euphorbia factor L1	Glioblastoma/In vitro and in vivo	[166]
SP94	Maleimide chemistry	Nanoprecipitation	RH40	PEG	≈145	Cryptotanshinone	Hepatocellular carcinoma/In vitro and in vivo	[167]
Penetratin, end-binding protein 1, MPG, and MPGΔNLS CPP	Carbodiimide and maleimide chemistries	Emulsification solvent evaporation	PVA	Avidin-palmitate/ DSPE-PEG	≈325 to 390	–	Cellular uptake enhancement/In vitro	[168,169]
Tat	Carbodiimide and maleimide chemistries	Emulsification solvent evaporation	Polysorbate 80	–	≈60	–	Cellular uptake enhancement/In vitro	[170]
CPPs (Tat, pAntp4, G2)	Physical adhesion	Nanoprecipitation	PVA	PEG	≈150 to 170	Fluorometholone	Ocular inflammatory disorders/In vitro and in vivo	[171]
CPPs, e.g., Tat, penetratin, and poly(arginine) 8	Physical adhesion	Emulsification solvent evaporation	PVA (31 kDa)	CS/PEG/Pluronic F127	≈150	–	Inner-ear therapy/In vitro and in vivo	[172]
CPPs (R8, Tat, penetratin), and a secretion peptide	Physical adhesion	Emulsification solvent evaporation	Sugar Ester S-1670	–	≈115 to 160	Insulin	Enhanced oral bioavailability of insulin/In vitro and in vivo	[173]
Angiopep-2	Maleimide chemistry	Nanoprecipitation	Pluronic^®^ F-127	PEG	≈165 to 180	–	Brain targeting/In vivo	[174]
Cyclic-RGD peptide	Maleimide chemistry	Double emulsion solvent evaporation	Pluronic^®^ F-127	PEG	≈310 to 330	–	Angiogenic endothelium targeting/In vitro	[175,176]
RGD peptides	Covalent conjugation to Pluronic^®^ F-127 via vinylsulfone-tiol reaction, and surface adhesion	Microfluidics-based nanoprecipitation	Pluronic^®^ F-127	PEG	≈140 to 160	–	Ovarian carcinoma and glioma/In vitro	[177]
Cyclo-(1,12)- PenITDGEATDSGC	Carbodiimide chemistry	Solvent displacement	Pluronic^®^ F-127	–	≈285 to 305	Doxorubicin	Lung cancer/In vitro	[178]
**Sialic acid**	Carbodiimide chemistry	Nanoprecipitation	Pluronic^®^ F-68	–	≈125	Lycopene	Kidney injury/In vitro	[179,180,181,182]
	Electrostatic adsorption	Emulsification solvent evaporation	PVA	CS	≈195	Doxorubicin	Non-Hodgkin’s lymphoma/In vitro and in vivo	[183]
	Carbodiimide chemistry	Nanoprecipitation	Pluronic^®^ F-68	Similopioid peptide (BBB-penetrating peptide)	≈180	Loperamide	Central nervous system targeting/In vitro and in vivo	[184]
	Binds to sialic acid	Emulsification solvent evaporation	PVA (30 to 70 kDa)			Doxorubicin/Phloretin	–	[185]
**Transferrin**	Physical adsorption	Emulsification solvent evaporation	PVA (30 to 70 kDa)	–	≈465	Paclitaxel/Superparamagnetic NP	Breast cancer, brain glioma/In vitro	[186,187]
	Carbodiimide chemistry	Emulsification solvent evaporation	PVA	–	≈210	Docetaxel	Breast cancer/In vitro	[188]
	Physical adsorption	Emulsification solvent evaporation	Pluronic^®^ F-127	–	≈200	Bortezomib	Pancreatic cancer/In vitro	[189]
	Physical adsorption	Double emulsion solvent evaporation	PVA	Lipid coat (lecithin/DSPE-PEG)	≈110	Doxorubicin	Lung cancer/In vitro and in vivo	[190]
	Carbodiimide chemistry	Nanoprecipitation	PVA	PEG	≈110	Thymoquinone	Lung cancer/In vitro and in vivo	[191]
	Carbodiimide chemistry	Emulsification solvent evaporation	PVA	PEG	≈150	Temozolomide	Brain glioma/In vitro and in vivo	[192]
	Physical adsorption	Nanoprecipitation	–	–	≈90	–	Brain glioma/In vitro and in vivo	[193]
	Carbodiimide chemistry	Double emulsion solvent evaporation	–	–	≈150	Doxorubicin/paclitaxel	Brain glioma/In vitro and in vivo	[194]

## Data Availability

No new data were created or analyzed in this study. Data sharing is not applicable to this article.
